# Flow invariant droplet formation for stable parallel microreactors

**DOI:** 10.1038/ncomms10780

**Published:** 2016-02-23

**Authors:** Carson T. Riche, Emily J. Roberts, Malancha Gupta, Richard L. Brutchey, Noah Malmstadt

**Affiliations:** 1 Mork Family Department of Chemical Engineering and Materials Science, University of Southern California, 925 Bloom Walk, HED 216, Los Angeles, California 90089, USA; 2 Department of Chemistry, University of Southern California, Los Angeles, California 90089, USA

## Abstract

The translation of batch chemistries onto continuous flow platforms requires addressing the issues of consistent fluidic behaviour, channel fouling and high-throughput processing. Droplet microfluidic technologies reduce channel fouling and provide an improved level of control over heat and mass transfer to control reaction kinetics. However, in conventional geometries, the droplet size is sensitive to changes in flow rates. Here we report a three-dimensional droplet generating device that exhibits flow invariant behaviour and is robust to fluctuations in flow rate. In addition, the droplet generator is capable of producing droplet volumes spanning four orders of magnitude. We apply this device in a parallel network to synthesize platinum nanoparticles using an ionic liquid solvent, demonstrate reproducible synthesis after recycling the ionic liquid, and double the reaction yield compared with an analogous batch synthesis.

## Abstract (web summary)

Microfluidic reactors offer cheap and simple solutions for nanoparticle synthesis, but suffer from the variation in particle size due to flow fluctuations. Riche *et al*. fabricate a device that is robust over a large range of flow rates and enables consistent particle synthesis in parallel operation.

## Graphical abstract

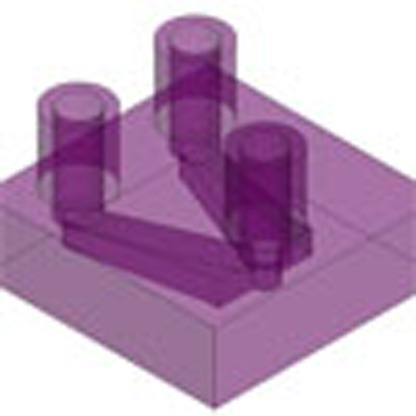

Continuous flow microfluidic reactors are powerful tools for synthesizing chemicals and materials^1^,^2^,^3^,^4^,^5^,^6^. Microreactors allow for efficient heat transfer, excellent control of local mixing conditions and provide an ideal format for studying reaction kinetics on a small scale^7^. Microfluidic systems also offer a clear and appealing route to scale-up via massively parallel operation. Scaling by parallelization has clear advantages over traditional scale-up approaches. In contrast to a scale-up approach that relies on increasing the size of a single batch reactor, scale-up by parallelization does not change the local reaction conditions in terms of mixing uniformity and temperature distribution, which are critically sensitive variables for certain chemistries. For example, the scale-up of colloidal inorganic nanoparticle syntheses to yield kg quantities is difficult to execute in conventional batch reactors because higher reagent concentrations or increased reaction volumes affect mass and thermal transport, which in turn affect nucleation and growth, leading to loss of particle quality and poor process reproducibility^8^. Microfluidic parallelization can circumvent these issues and enable a simplified and more predictable scale-up route, as demonstrated in a parallel network of planar droplet formation devices^9^.

One major challenge in implementing parallel microreactor systems is developing control and design strategies that guarantee uniform fluidic behaviour across an ensemble of reactors^10^,^11^. Here, we address this issue by presenting a fluidic design based on a three-dimensional (3D)-printed channel junction that allows for geometrically controlled two-phase liquid-in-liquid droplet formation that is robust to fluctuations in driving pressure and flow rate. Microfluidic liquid-in-liquid droplet flows facilitate rapid homogenization of reactants^12^. They are, therefore, ideal for reactions that are sensitive to concentration gradients and local mixing conditions. Droplets are isolated from each other and the channel walls to eliminate dispersion effects and prevent device fouling^13^. The design presented here enables an ensemble of parallel reactors with consistent droplet formation behaviour regardless of inconsistencies in the feed pressure or flow rate across the reactor bank. In contrast, prior attempts at nanoparticle synthesis scale-up using continuous flow have focused on modifying single-channel devices to employ larger droplets and increased operating flow rates^14^,^15^. Although these approaches produce good quality nanoparticles, this strategy cannot be scaled indefinitely.

An additional advantage of the droplet formation geometry we present here is that it can be rapidly reconfigured to produce a variety of droplet volumes spanning four orders of magnitude. This range of droplet sizes is used in many applications, including biomimetic vesicle formation, cell encapsulation and millifluidic reactor platforms^16^,^17^. In traditional T-junction and flow-focusing droplet formation devices, the operating parameters (that is, flow rates) allow for a relatively narrow range of droplet sizes to be accessed by a single device geometry^18^. Switching to a different droplet size regime requires redesigning (and refabricating) the device. In the device geometry we present here, droplet size is set by the diameter of an easily interchangeable outlet component. Different droplet sizes are accessible by swapping out this modular component. This control mechanism is in contrast to upstream geometrical control exhibited in planar droplet formation devices where the inlet geometry governs droplet size. Coupled with the relative insensitivity of droplet formation to flow rates, this design represents an important innovation in microfluidic droplet formation.

In addition to demonstrating the robust operation of this droplet formation geometry in a parallel system, we show it operating as the key element of a droplet microreactor applied to the synthesis of metal nanoparticles. In this paper, we demonstrate the first platinum nanoparticle (PtNP) synthesis using a continuous flow droplet microreactor. A key aspect of our continuous flow synthesis is the use of ionic liquid droplets as the dispersed phase and reaction medium. Ionic liquids are gaining interest as solvents for precious metal nanoparticle synthesis because of their ability to colloidally stabilize nanoparticles and induce high nucleation rates resulting in more monodisperse nanoparticle ensembles^19^. This, coupled with their environmental health, safety and sustainability advantages over volatile and flammable organic solvents^20^, makes ionic liquids promising solvents for large-scale nanofabrication reactions. Herein, the synthesis of PtNPs is performed in ionic liquid droplets that are successfully recycled and reused to produce PtNPs over multiple runs in high fidelity.

## Results

### 3D-printed microfluidic droplet generators

**Figure 1:**
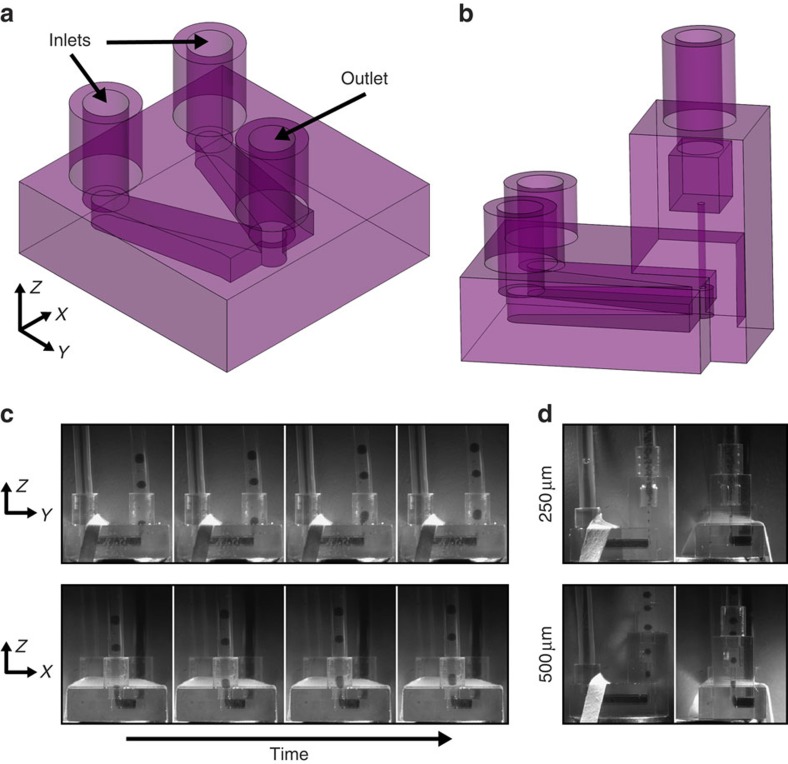
Renderings of 3D droplet generators and images of droplet formation process. (**a**) Computer-aided design (CAD) rendering of a droplet generator with two inlets for the dispersed and continuous phases and a single outlet that accepts tubing (OD=1/16 inch) with various IDs to control the droplet size. (**b**) CAD rendering of a droplet generator in which the vertical segment is fully constructed by stereolithography (SLA) rather than being formed by external tubing. (**c**) Micrographs depicting different views of the device during the droplet breakup process. (**d**) Micrographs of the droplet breakup process in full SLA droplet generators with an outlet size of 250 or 500 μm.

This microfluidic droplet generator is designed to be an easy-to-operate device that forms consistent droplet sizes across a broad range of inlet pressures or flow rates. The 3D geometry is manufactured using stereolithographic (SLA) printing technologies. This geometry is designed to interface with commercially available tubing (outer diameter (OD)=1/16 inch) to create a droplet generating chip that does not require any fabrication steps in a clean room facility and can passively form droplet volumes spanning four orders of magnitude. Tubing connections form an elastomeric seal that resists leaking for water flow rates beyond 8 l h^−1^. Although the 3D printing technology is quickly advancing to create higher resolution features, a major concern is that the smallest channel feature that has been produced in the commonly used Watershed material is 400 μm. Herein, we overcome this barrier by (i) fabricating a device with a 250-μm feature and (ii) using the printed device as a fluidic manifold while relying on higher resolution extruded tubing to control droplet size. The channels deliver the laminated dispersed and continuous phases to the outlet in a perpendicular orientation (Fig. 1a,b). Droplets pinch off as the fluids enter the vertical outlet tubing (Fig. 1c,d). The entire droplet formation process can be seen in the Supplementary Movie 1.

**Figure 2:**
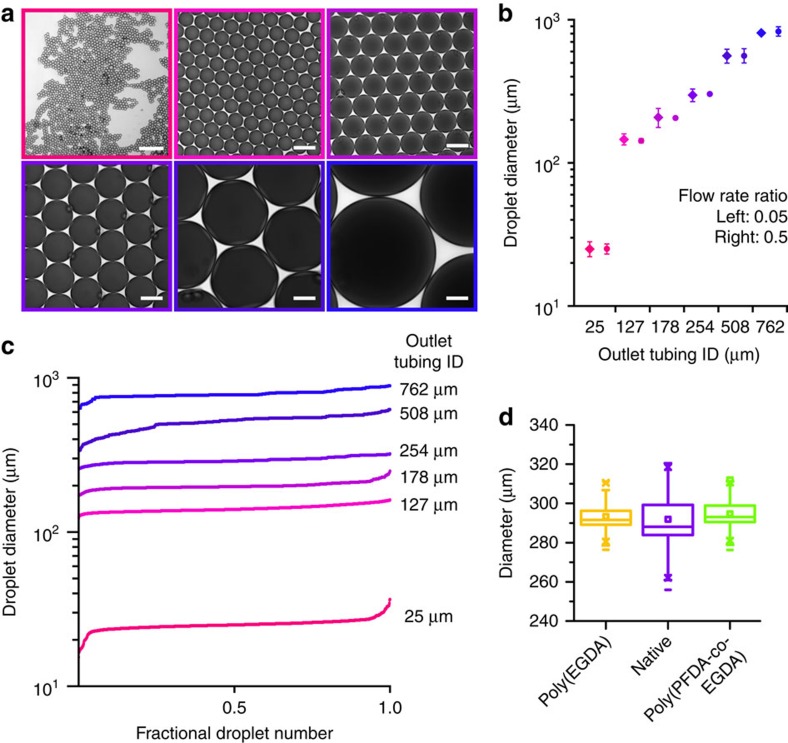
Images of droplets and statistics on droplet sizes using various outlet tubing sizes. (**a**) Micrographs of the droplets formed using the six different sizes of outlet tubing listed. (**b**) Droplet diameter versus outlet tubing inner diameter for flow rate ratios of 0.05 (left, diamond) and 0.5 (right, circle). Error bars represent the s.d., some error bars are obscured by the symbols. (**c**) Plot of the droplet diameter versus fractional droplet number for various outlet tubing sizes. The solid lines represent the average droplet sizes for a single outlet size; each line includes values for flow rate ratios of 1:2 and 1:20 (dispersed to continuous phase). (**d**) Boxplot of the droplet size produced by droplet generators with the same outlet tubing (ID=254 μm) and different surface chemistries (that is, hydrophilic, native and hydrophobic) on the channels, as modified by initiated chemical vapour deposition.

The basic 3D-printed device shown here can be used to form a broad range of droplets sizes by interfacing it with outlet tubing of various inner diameters. The size of the outlet tubing determines the droplet size such that the droplet sizes are similar to the inner diameter of the outlet tubing (Fig. 2a). In comparison, a planar T-junction geometry produces droplets with a size governed by the geometry of the dispersed phase inlet^21^. Regardless of the outlet tubing size, the droplet population is monodisperse, which allows for the collected droplets to self-assemble into hexagonally close packed arrays (Supplementary Figs 1–3).

We also present a droplet generator where the vertical cavity is integrated into the SLA manufactured device rather than being provided by external tubing (Fig. 1b,d). The breakup process is imaged in fully printed devices with cylindrical sizes of inner diameter (ID)=250 and 500 μm (Supplementary Movie 2). We observe droplets forming at the point where the horizontal flow turns vertical, as in the droplet generators with externally connected tubing acting as the vertical cavity. As the dispersed and continuous phases enter the vertically oriented cylinder, the dispersed phase segments into droplets. This 250 μm cylinder is the smallest channel reported using the SLA process with transparent Watershed resin (FineLine Prototyping)^22^. Post printing, FineLine Prototyping clears unreacted resin from the vertical cavity and the devices are used as received.

### Flow invariant droplet formation

The defining characteristic of this droplet-forming device is that uniform droplets can be formed while varying the flow rate ratio. We demonstrate flow invariant droplet formation for six different commercially available sizes of outlet tubing. The smallest and largest inner diameters are 25 and 762 μm, respectively. For each tubing size, flow invariant droplet formation is observed up to an upper limit flow rate (Fig. 2b). By analysing the sizes of collected droplets, we determine the upper limit of the flow invariant regime for each tubing size. The data presented are collected at this upper limit for the continuous phase flow rate of 0.2, 5, 10, 20, 80 and 180 ml h^−1^ corresponding to the outlet tubing with an inner diameter of 25, 127, 178, 254, 508 and 762 μm, respectively. Below this upper limit, the droplet size is approximately the same as the diameter of the outlet tubing (Fig. 2b).

The upper limit of the flow invariant regime can be expressed in terms of the capillary number (*Ca*) of the system. For all outlet tubing sizes, this value (calculated using the inner diameter of the outlet as the characteristic length and the continuous phase flow rate as characteristic velocity) is about 10^−3^. Below this capillary number, the droplet size is independent of the flow rate ratio for values of 1:20 and 1:2 (Fig. 2c). The droplet diameters are plotted versus the fractional droplet number. These data show the entire population of droplets. The total number of droplets is normalized to one and represented as a fractional droplet number. We also observe a consistent droplet size for intermediate flow rate ratios (Supplementary Fig. 4). At higher capillary numbers, in the flow-dependent regime, the droplet formation process transitions to a jetting mechanism (Supplementary Movie 3). The same flow invariant behaviour is observed for a more viscous dispersed phase of 70 wt% glycerol in water (Supplementary Fig. 5). However, the threshold for invariance shifted to a lower capillary number. In the flow invariant regime, the median droplet sizes have a coefficient of variation <3%.

The output of the droplet generator is dependent on the geometry of the outlet tubing and not dependent on the surface chemistry of the channel before the outlet. We modify the internal channel surfaces by depositing a poly(ethylene glycol diacylate) or poly(1*H*,1*H*,2*H*,2*H* perfluorodecylarcylate-co-ethylene glycol diacrylate) polymeric film via initiated chemical vapour deposition (iCVD). The two coatings have water contact angles of 60° and 120°, respectively. The native (uncoated) material has a contact angle of 100°. With the same outlet tubing (ID=254 μm), the coated devices produce the same size droplets as the unmodified device (Fig. 2d). The roughness of the channel surfaces was the same before and after coating by scanning electron microscopy analysis (Supplementary Fig. 6).

### Device parallelization

A single-droplet formation device can be used as a single unit in a highly parallelized system of *n* units to linearly create *n*-fold droplets and *n*-fold throughput. Ideally, a parallelized system of single-droplet generators is designed to have an equal pressure drop and resistance over each channel to create identical flow conditions in each droplet generator. However, feedback between channels arises because of unequal numbers of droplets flowing in each channel^23^. This imbalance leads to flow rate fluctuations that alter the final droplet size, but the fluctuations are irrelevant in the 3D droplet generators because of their insensitivity to flow rate. For this reason, our droplet generator is uniquely suited for use in a parallelized network.

**Figure 3:**
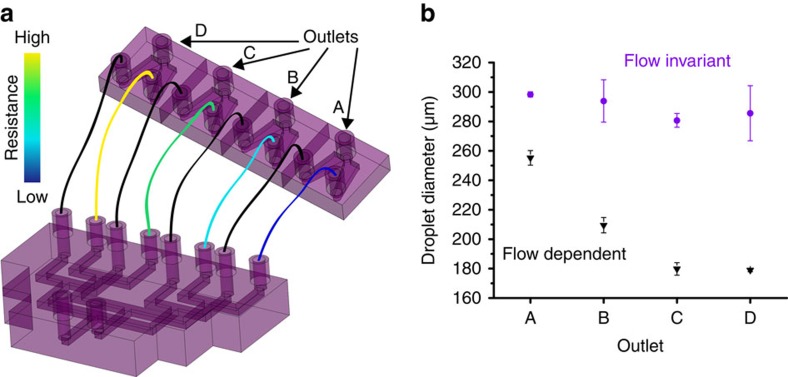
Setup and performance of unbalanced parallel network. (**a**) Schematic of the parallel network assembled by connecting a distribution manifold to four droplet generators. The continuous phase was linked using low resistance jumper tubing (ID=762 μm) and the dispersed phase was linked using various lengths of tubing (ID=127 μm) to create a gradient of resistances across the four branches. (**b**) Droplet diameters (*n*>1,000) produced by the four branches of the parallel network (left) by dispersed and continuous phase flow rates of 10 and 70 ml h^−1^ (purple circles) and 30 and 210 ml h^−1^ (black triangles) while operating in and beyond the flow invariant regime, respectively. Error bars represent the s.d.

To demonstrate this, we construct a parallel network (*n*=4) of droplet generators and deliver different flow rates to each device (Fig. 3a). We print a manifold to distribute the dispersed and continuous phases to four droplet generators. The manifold connects to four independent droplet generators by jumper cables (that is, sections of poly(ether ether ketone) (PEEK) tubing). The jumper cables connecting the dispersed phases have lengths of 10, 12.5, 15 and 17.5 mm, resulting in relative resistances of 1x, 1.25x, 1.5x and 1.75x, respectively, because the resistance is linearly proportional to the length of cylindrical tubing. The network assembly creates the largest pressure drop over the dispersed phase jumper cables so there is minimal feedback from the continuous phase jumper cables or the outlets. The ID of the jumper cables are 127, 762 and 254 μm for the dispersed phase, continuous phase and outlets, respectively.

**Figure 4:**
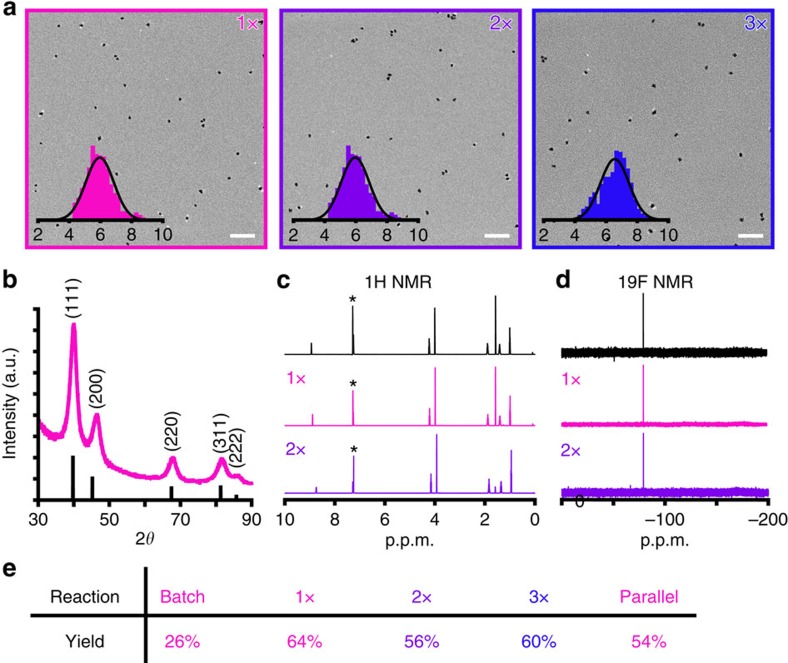
Micrographs and characterization of PtNPs synthesized in ionic liquid solvent using 3D droplet generator. (**a**) Transmission electron microscopy (TEM) images of PtNPs produced using the microfluidic droplet generator using 1x recycled, 2x recycled and 3x recycled BMIM-Tf_2_N ionic liquid. Scale bars represent 50 nm and histograms represent the NP diameters (*n*=500) from multiple TEM images. (**b**) X-ray diffraction (XRD) of the PtNPs. (**c**) ^1^H NMR spectra of the BMIM-NTf_2_ ionic liquid (*residual solvent peak), and (**d**) ^19^F NMR spectra of the BMIM-Tf_2_N ionic liquid. Both spectra in black are of the ionic liquid as received. (**e**) Comparison of the overall yield from various reactions.

The network successfully delivers the same continuous phase flow rate and different dispersed phase flow rates to each droplet generator. When operating outside the flow invariant regime (that is, at high capillary number), the droplet size produced is dependent on the branch location and therefore the dispersed phase flow rate (Fig. 4b). As expected, the droplet size is smaller in branches with a lower dispersed phase flow rate. In contrast, when operating within the flow invariant regime (that is, at low capillary number), the droplet size is independent of the branch location despite a different dispersed phase flow rate being delivered to each channel (Fig. 4b). As expected, the balanced parallel network delivers the same flow rates to each droplet generator and produces a constant droplet size across each of the devices, in both flow regimes (Supplementary Fig. 7).

### PtNP synthesis

The droplet generator presented here is suitable for chemical synthesis in dispersed phases with a wide range of solvent properties. As a proof-of-concept, we demonstrated the synthesis of PtNPs by a polyol reduction in droplet flows of 1-butyl-3-methylimidazolium bis(trifluoromethylsulfonyl)imide (BMIM-Tf_2_N) ionic liquid solvent. There are few examples of droplet flows of ionic liquids because they represent an exceptional case of droplet flow behaviour as a result of their complex interfacial properties and high viscosity^1^,^24^,^25^. Here, BMIM-Tf_2_N ionic liquid droplets are formed trivially in a modified droplet generator with three inlets to accommodate two reagent/dispersed phase streams (Supplementary Fig. 8). The two reagent inlets supply (i) the potassium tetrachloroplatinate(II) (K_2_PtCl_4_) precursor and the reducing agent (that is, ethylene glycol) and (ii) the poly(vinylpyrrolidone) (PVP) in BMIM-Tf_2_N. Droplets of the combined reagents are formed using PEEK tubing (ID=762 μm) in the outlet. The reaction is initiated by flowing the droplets into a convection oven at 150 °C to quickly nucleate the PtNPs. The temperature in the tubing equilibrates in less than a second to trigger the nucleation event. Likewise, an abrupt cooling step quickly quenches the reaction and arrests nanoparticle growth. There is no observable clogging of or deposition on the channel surfaces after a 2-h reaction (Supplementary Fig. 9).

Powder X-ray diffraction analysis confirms the resulting nanoparticles crystallize in the face centered cubic structure expected for Pt metal. An average lattice parameter of *a*=3.87 Å is calculated for the PtNPs, which is in close agreement with bulk Pt metal (PDF# 00-004-0802). Moreover, the diffraction peaks are broadened, suggesting the presence of small nanoparticles on the order of ∼6 nm by the Scherrer equation (Fig. 4b). We synthesize three rounds of PtNPs and recycle the BMIM-Tf_2_N ionic liquid solvent between rounds. To recycle the ionic liquid solvent, the PtNPs are harvested from the ionic liquid and then it is washed to remove excess ethylene glycol and PVP. The purity of the BMIM-Tf_2_N is unchanged, as evidenced by ^1^H and ^19^F nuclear magnetic resonance (NMR), upon recycling (Fig. 4c,d and Supplementary Note 1). Transmission electron microscopy images reveal that the PtNPs appear spherical and uniform in morphology across each reuse of the ionic liquid (Fig. 4a). For each condition, 500 particles are analysed and their average sizes are 5.65±0.76, 5.96±0.90 and 6.56±0.92 nm for 1x, 2x and 3x recycled ionic liquid, respectively. The mean sizes are all within the standard deviation of each other.

We provide a general device and platform for running reactions in parallelized droplet reactors. When PtNPs are synthesized in an *n*=4 parallel network, all four branches produce high-quality particles, as evidenced by consistent size distributions that are within error of each other (Supplementary Fig. 10). In addition, translation of the PtNP synthesis from batch scale to a continuous flow droplet reactor results in significantly higher yields (Fig. 4e). For each run with the recycled ionic liquid and the overall parallel reaction, the continuous flow yield is about twice the batch yield. We attribute the greater yield to a more rapid and uniform heating profile, as well as improved mixing as compared with the batch reaction. The combined use of our droplet generator in a parallel network and a reusable solvent system provide an ideal platform for the efficient manufacturing of large quantities of nanomaterial product.

## Discussion

We introduce a novel droplet generator that uses a 3D geometry to form droplets of controlled size. A key feature of this droplet formation geometry is that there is a broad regime of inlet flow rate ratios over which resulting droplet size is invariant to flow rate. Another advantage of this droplet formation format is that its inherent modularity makes it simple to select the size of droplets that will be formed. The size can easily be tuned by changing the ID of the outlet tubing. The ease of device fabrication and operation lowers the barrier-to-entry for first time users of microfluidic devices. An end user need not have an extensive fluid mechanics understanding to operate the device to achieve the desired and well-controlled droplet sizes.

The relationship between outlet tubing size and droplet size seems to underlie droplet size stability. We speculate that this relationship is due to the mode of droplet formation. Droplet breakup occurs as the two flows alternate to enter and fill the opening of the vertical cavity created by the outlet tubing. Droplets form at the interface between the outlet tubing and the horizontal flow when the dispersed phase is pinched off to form droplets (Fig. 1c,d and Supplementary Movie 1). When the dispersed phase fills the outlet it causes an upstream buildup of continuous phase pressure. Once the outlet is completely occluded by the dispersed phase, the droplets shear off, releasing the continuous phase pressure. As outlet tubing size increases, more dispersed phase accumulates before the continuous phase is completely occluded, explaining the correlation between outlet tubing size and terminal droplet size.

These droplet-forming devices are uniquely suited to high-throughput processing using microfluidics. When assembled in a parallel configuration, they are insensitive to small changes in flow that could arise because of feedback between channels. This is demonstrated by building a four-branched parallel network that produces droplets of similar size across the network despite having an intentional gradient of dispersed phase flow rates being delivered to each branch. The devices also resist clogging that could affect droplet formation.

We synthesize monodisperse PtNPs over multiple runs while using the same recycled ionic liquid solvent. The yield in the continuous flow reactions is ∼60% and nearly twice the yield of an analogous batch reaction. Using this device infrastructure along with more sustainable chemistry provides an ideal platform for producing large quantities of precious metal nanoparticles. This can be easily extended to other applications requiring high-throughput synthesis in microfluidic droplets.

## Methods

### Device fabrication

Microfluidic chips were designed in ProEngineer, exported as sterolithography files and printed in Somos Watershed XC 11122 by FineLine Prototyping using high-resolution SLA printing technology. Devices were used as received. Inlet and outlets interfaced with OD=1/16 inch tubing. The device shown in Fig. 1a has a channel height of 1 mm, inlet and outlet holes were 1.59 mm in diameter, the length of the main channel was 5 mm and the width of the main channel was 4 mm. The fully 3D printed droplet generator in Fig. 1b has the same dimensions as the device in Fig. 1a and the additional vertical cavity was printed with an internal diameter of 250 or 500 μm.

### Droplet visualization

Water-in-oil droplets were formed using an aqueous phase of Fe(SCN)_x_^(3-x)+^ complex (for visualization) in deionized water, prepared by mixing 0.2 M KSCN (Sigma) with 0.067 M Fe(NO_3_)_3_·9H_2_O (Sigma) in a 1:1 volumetric ratio. The oil phase was 1% (w/v) Span80 (Sigma) in hexanes (Sigma). Fluids were driven by syringe pumps (Harvard Apparatus). Droplets were collected in a glass bottom Petri dish (MatTex) containing 1 ml of the 1% (w/v) Span80 in hexanes solution and imaged on an inverted Zeiss microscope using a × 20 objective. At least 60 images of the collected droplets were captured for each flow condition. Droplet sizes were analysed using custom image processing code in Matlab, primarily using the imfindcircles function.

Droplet formation was monitored *in situ* using a Phantom V711 camera (Vision Research). Images were captured at 4,000 frames per second with a 240-μs exposure.

### PtNP synthesis in microfluidic device

K_2_PtCl_4_ (99.9%; Strem), PVP (MW=55,000; Aldrich), ethylene glycol (99.8%; Sigma-Aldrich) and BMIM-Tf_2_N (99%; IoLiTec Lot # K00219.1.4.) were used as received. In a typical procedure, K_2_PtCl_4_ (156.0 mg) was added to ethylene glycol (10.0 ml) and bath sonicated until dissolved, affording a brown-red mixture. Separately, PVP (852.4 mg) was dissolved in BMIM-Tf_2_N (30.0 ml) by heating at 130 °C for 10 min to give a clear solution.

PtNPs were synthesized using our 3D droplet-forming device by modifying the original design to incorporate three inlets (Supplementary Fig. 8). Two inlets supplied the K_2_PtCl_4_ in ethylene glycol at 10 ml h^−1^ and the PVP in BMIM-Tf_2_N at 30 ml h^−1^. The third inlet supplied a continuous phase of FC-40 (Sigma) at 90 ml h^−1^. The outlet PEEK (ID=0.03 inches) fed into 200 feet of perfluoroalkoxy tubing (McMaster-Carr) that was placed in a convection oven set to 150 °C. The effluent was collected into a receiving flask cooled in an ice bath to quench the reaction.

The PtNPs were transferred to two 50-ml centrifuge tubes and precipitated with acetone (30 ml) to afford a black suspension, which was briefly vortex stirred (∼1 min), bath sonicated (3 min) and collected by centrifugation (6,000 r.p.m.; 5 min). The colourless supernatant was decanted and saved in order to wash the ionic liquid for further syntheses. The black nanoparticulate solid was redispersed in ethanol (10 ml), bath sonicated (3 min), precipitated with hexanes (30 ml) and centrifuged (6,000 r.p.m.; 5 min). This process was repeated 3 × in order to remove any residual organics (that is, PVP, BMIM-Tf_2_N, and ethylene glycol). The purified PtNPs were re-dispersed in ethanol (5 ml) and remained colloidally stable for at least 5 months.

### Recycling the ionic liquid

Equal volumes of hexanes, with respect to the ionic liquid, were added and vortex stirred before centrifugation (6,000 r.p.m.; 10 min). Upon phase separation, the hexanes layer was removed and additional hexanes were added; this extraction procedure was repeated 3 × . The ionic liquid was dried in a rotary evaporator at 80 °C for ca 2 h. The purity of the washed ionic liquid was confirmed by ^1^H and ^19^F NMR spectroscopy.

### Initiated chemical vapour deposition

Devices were coated as received with poly(1*H*,1*H*,2*H*,2*H*-perfluorodecyl acrylate-co-ethylene glycol diacrylate) (poly(PFDA-co-EGDA)) or poly(ethylene glycol diacrylate) (poly(EGDA))^1^,^26^,^27^. Briefly, the devices were coated using the iCVD process. Microfluidic reactors were placed on a temperature-controlled stage maintained at 30 °C within a pancake-shaped vacuum chamber (GVD Corp., 250 mm diameter, 48 mm height). The polymer coating was formed via a free-radical chain mechanism from the reaction of vapour phase precursors. Di-*tert*-butyl peroxide (Sigma) and PFDA (SynQuest) and/or EGDA (Monomer-Polymer) were introduced into the vacuum chamber at a pressure of 100 mTorr and the initiator molecules were thermally decomposed into free radicals at 250 °C by a resistively heated array of nichrome wire. The radicals and monomer molecules diffuse into the channels via the inlets and outlet, adsorb to the surface of the material, and polymerize to form a thin, conformal coating.

### PtNP synthesis in batch

In a typical procedure, K_2_PtCl_4_ (39.0 mg) was added to ethylene glycol (2.5 ml) in a 23-ml vial and bath sonicated until dissolved, affording a brown-red mixture. Separately, PVP (213.1 mg) was dissolved in BMIM-Tf_2_N (7.5 ml) by heating at 130 °C for 10 min to give a clear solution. The two solutions were then combined in a 25-ml round bottom flask and placed in a silicone oil bath preheated to 150 °C while stirring at 1,000 r.p.m. The reaction was quenched with an ice bath after 15 min. The washing of the particles and the recycling of the ionic liquid followed the same procedure as in the microfluidic device reaction.

### Characterization

Transmission electron microscopy images were obtained using a JEOL JEM2100F (JEOL Ltd.) microscope operating at 200 kV. Samples were prepared on 400 mesh Cu grid coated with a lacey carbon film (Ted Pella, Inc.) by drop-casting a dilute suspension of PtNPs in ethanol. The size distribution of the PtNPs was determined by analysing 500 unique nanoparticles. Powder X-ray diffraction patterns were collected on a Rigaku Ultima IV diffractometer functioning at 40 mA and 40 kV with a Cu *K*α X-ray source (*λ*=1.5406 Å). The step size and collection time were 0.015° and 10 s per step, respectively. ^1^H and ^19^F NMR spectra were obtained on Varian 600 spectrometer (600 and 564 MHz, respectively) with chemical shifts reported in p.p.m.

## Additional information

**How to cite this article:** Riche, C. T. *et al*. Flow invariant droplet formation for stable parallel microreactors. *Nat. Commun.* 7:10780 doi: 10.1038/ncomms10780 (2016).

## Supplementary Material

Supplementary Information Supplementary Figures 1-10 and Supplementary Note 1

Supplementary Movie 1 The left and right movies show the back and side view, respectively, of a droplet generator with transparent PTFE ID = 762 μm tubing in the outlet. The dispersed and continuous phase flow rates are 22.5 and 90 mL h^-1^.

Supplementary Movie 2 The movies show fully printed 3D droplet generators containing vertical cavities with an ID = 250 μm (left) and ID = 500 μm (right). The dispersed and continuous phase flow rates are 5 and 20 mL h^-1^ (left) and 10 and 40 mL h^-1^ (right).

Supplementary Movie 3 The movie shows droplets forming outside the flow invariant regime, when jetting occurs. The outlet tubing is PTFE ID = 762 μm.
