# Supplementary material for: Flow invariant droplet formation for stable parallel microreactors
Source: Nat Commun. 2016 Feb 23;7:10780. doi: 10.1038/ncomms10780 (PMC4766398; doi:10.1038/ncomms10780)
Supplement: Supplementary Information — Supplementary Figures 1-10 and Supplementary Note 1 [file ncomms10780-s1.pdf]

Supplementary Figures

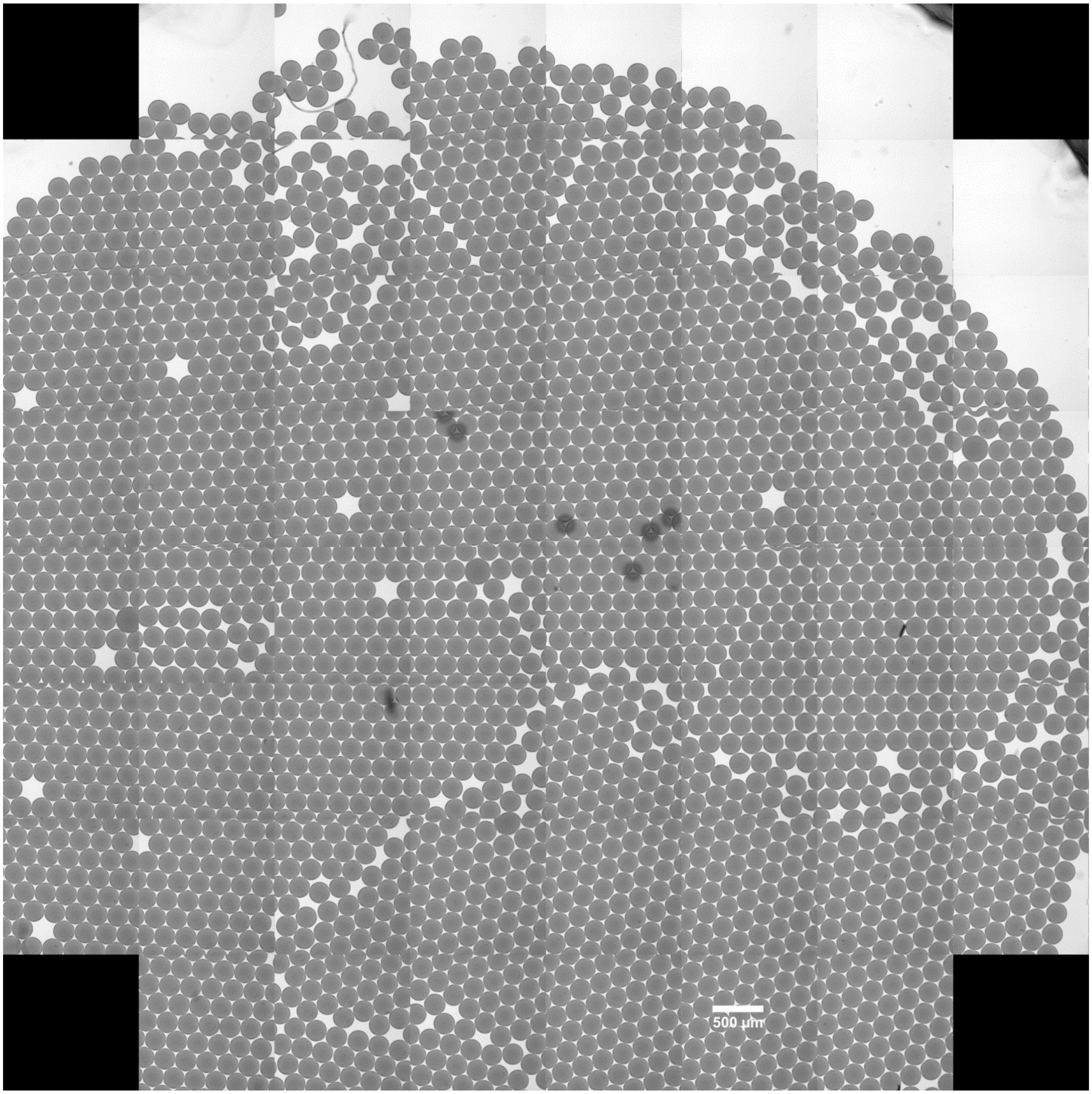

Supplementary Figure 1 Droplet images. 60 micrographs of droplets produced using PEEK tubing in the outlet (ID = 178 µm). Scale bar represents 500 µm.

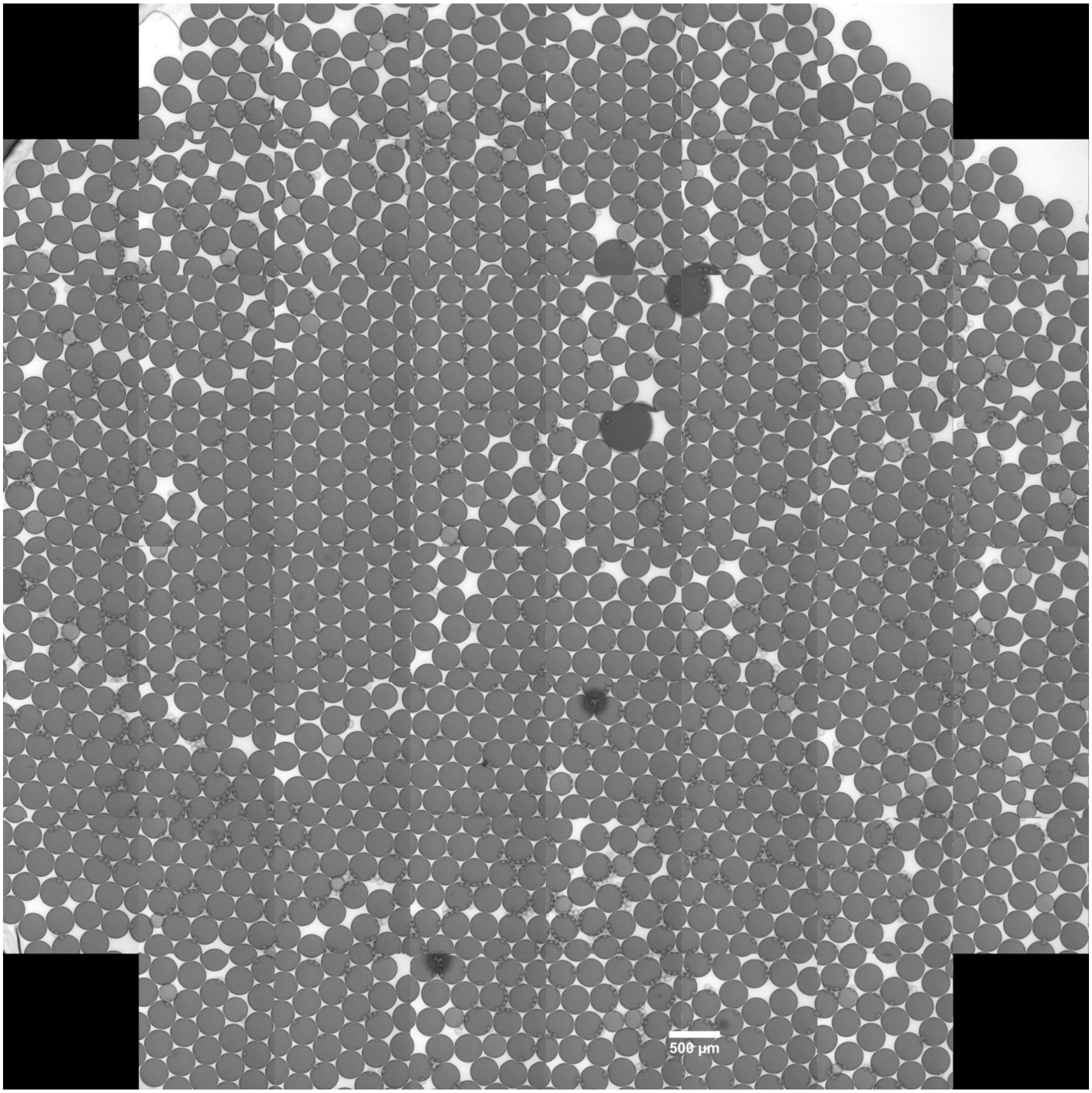

Supplementary Figure 2 Droplet images. 60 micrographs of droplets produced using PEEK tubing in the outlet (ID = 254 µm). Scale bar represents 500 µm.

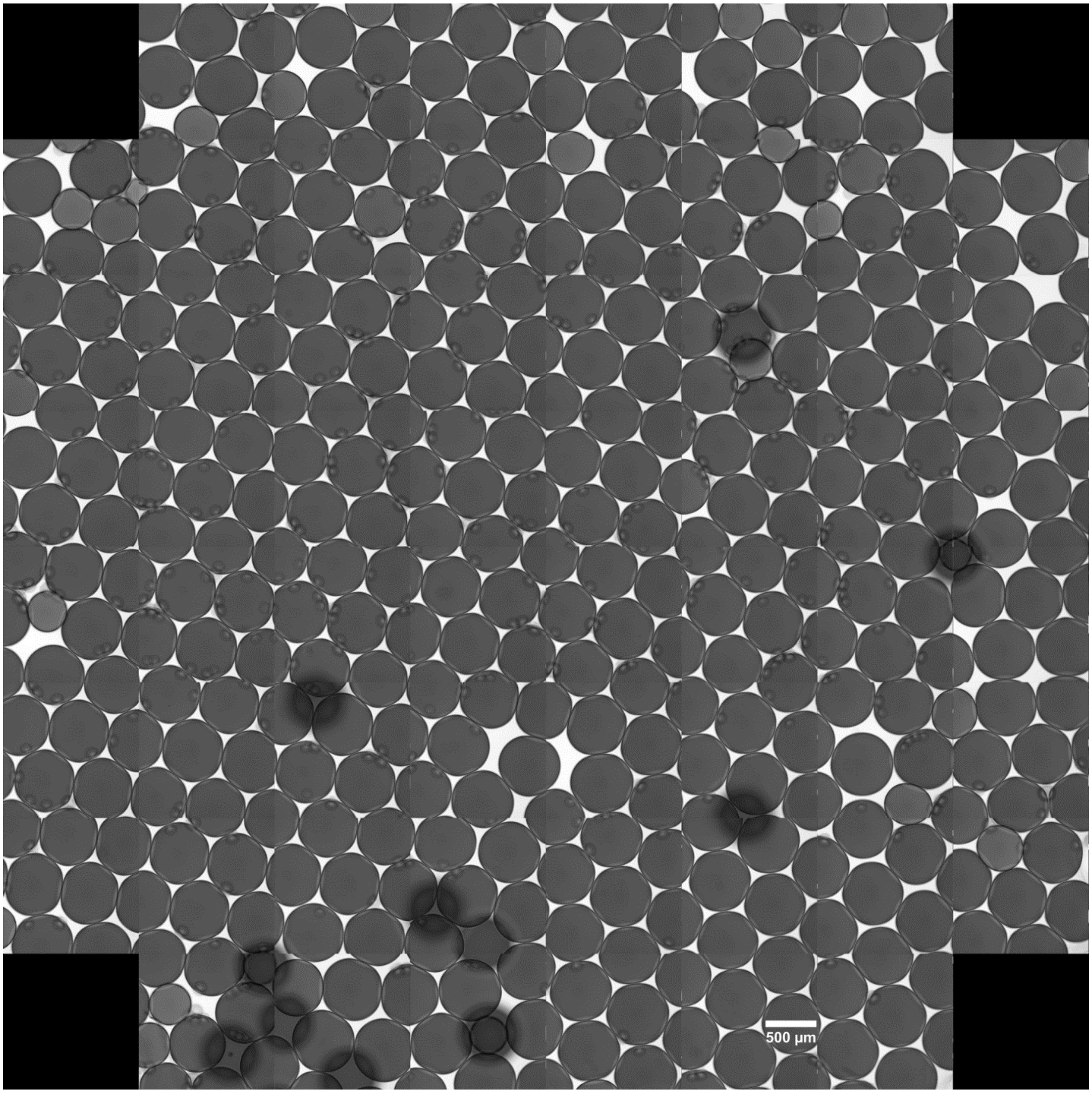

Supplementary Figure 3 Droplet images. 60 micrographs of droplets produced using PEEK tubing in the outlet (ID = 508 µm). Scale bar represents 500 µm.

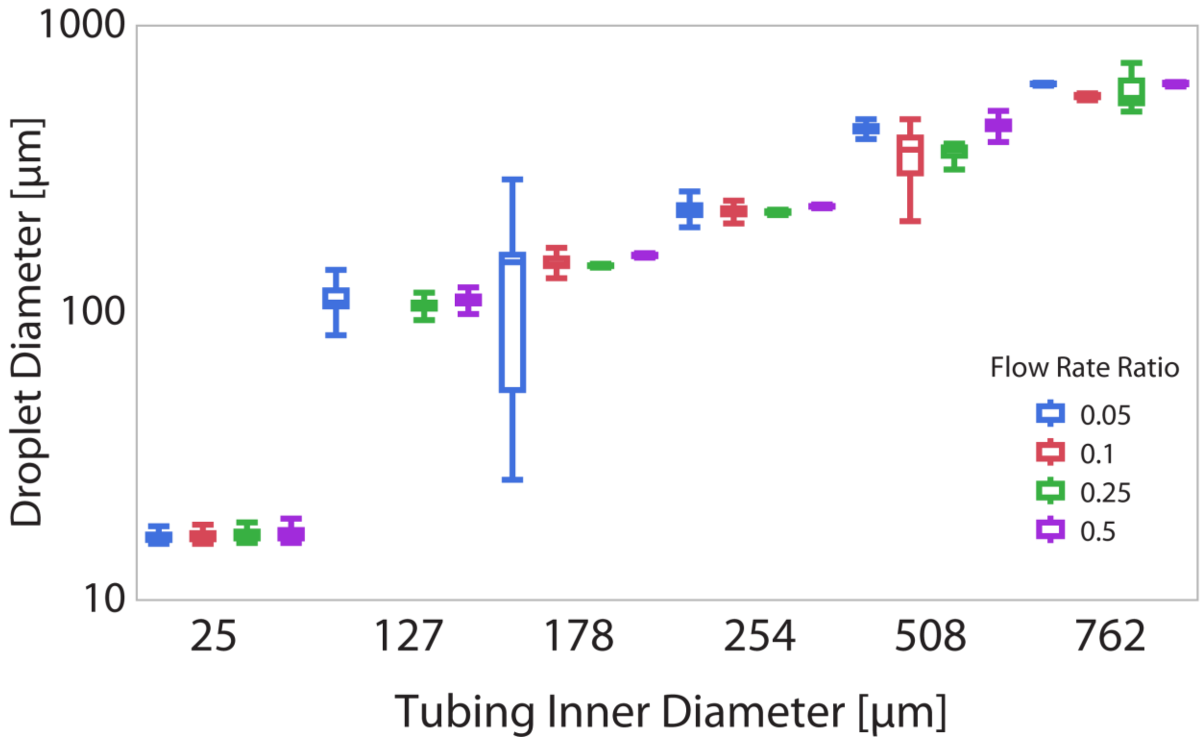

Supplementary Figure 4 Statistics for droplet sizes. Boxplot of the droplet sizes produced for different sizes of outlet tubing and different flow rate ratios.

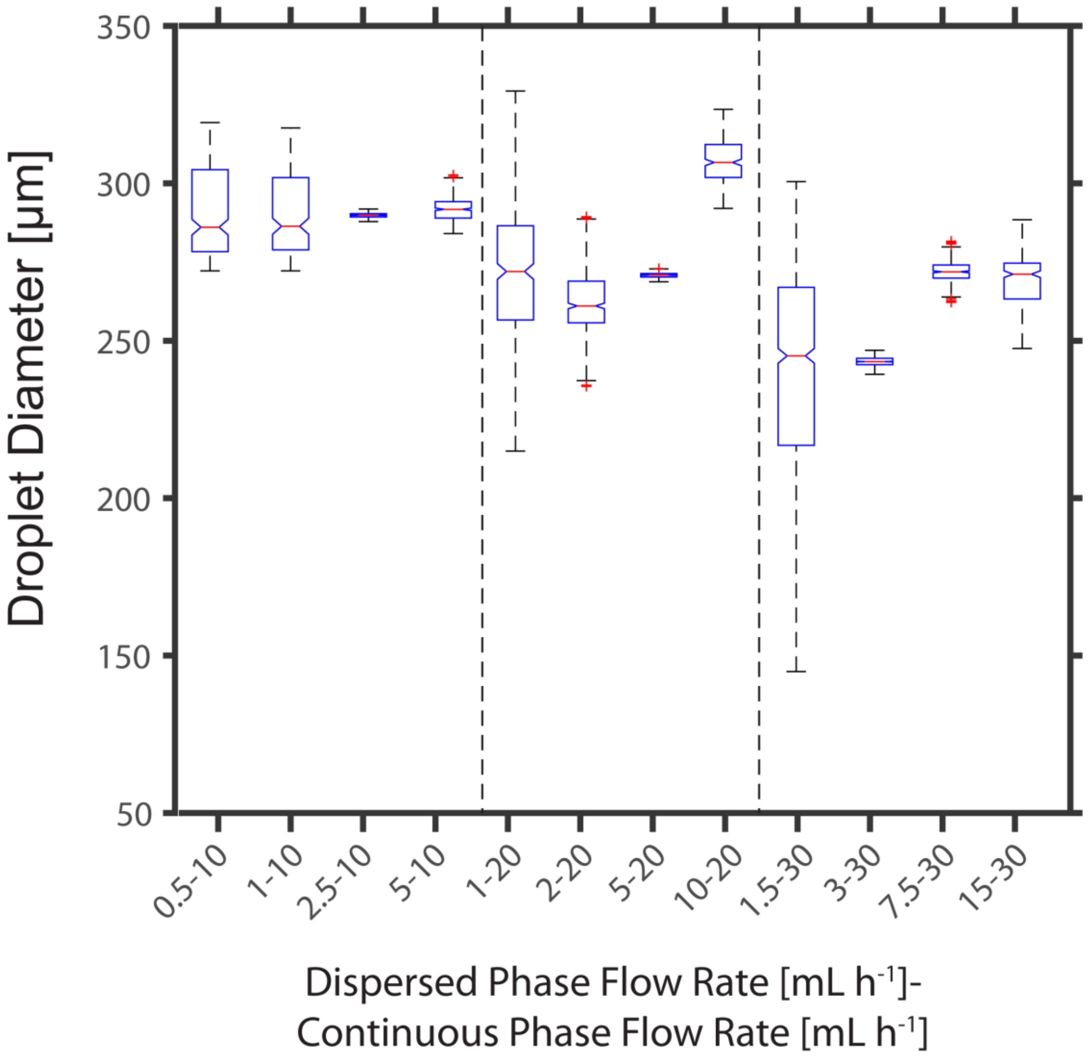

Supplementary Figure 5 Statistics for viscous droplets. Boxplot of glycerol droplets formed using the 3D droplet generator and PEEK outlet tubing (ID = 254 µm).

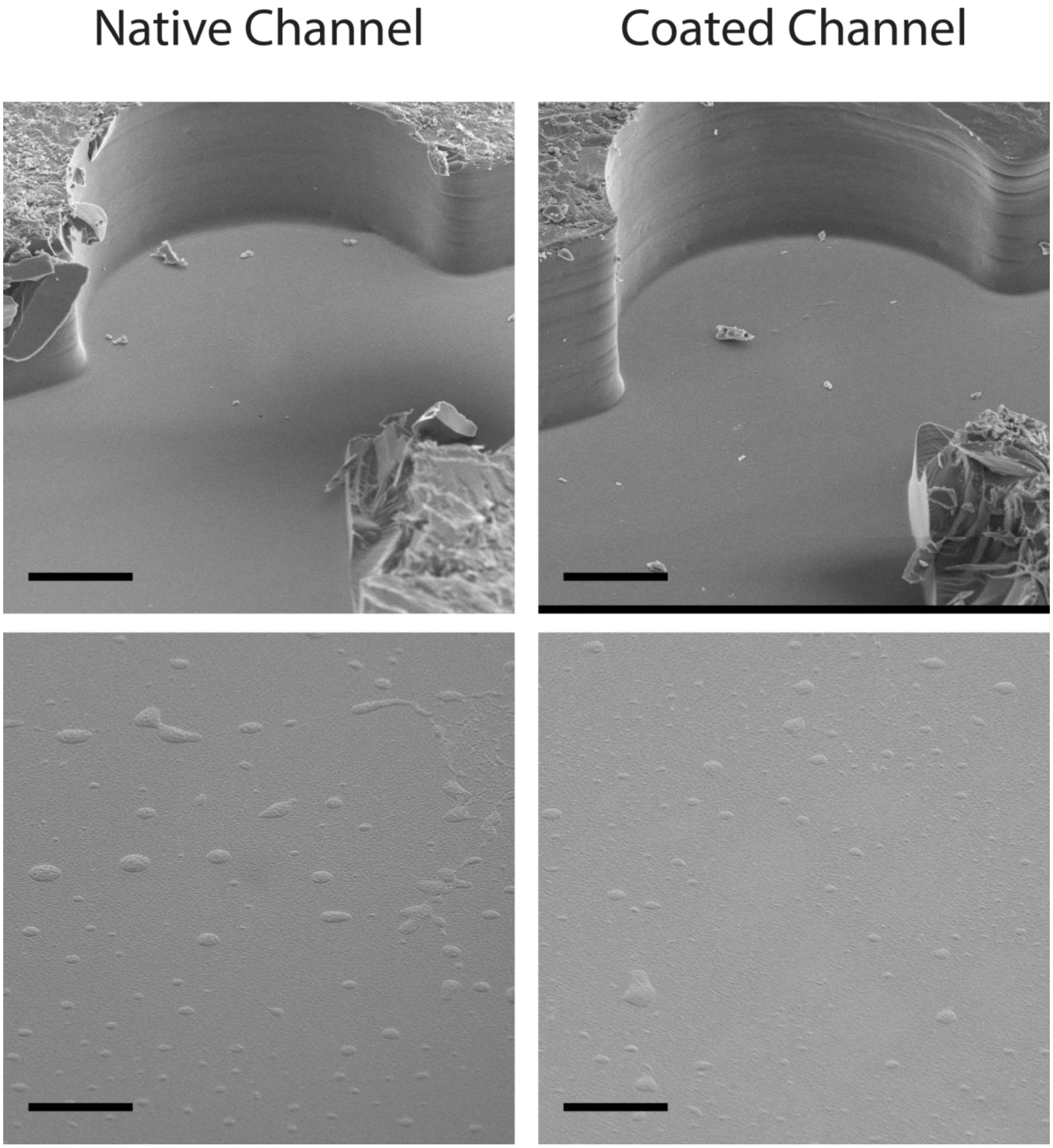

Supplementary Figure 6 Comparison of uncoated and coated channels. SEM micrographs of the channels within an uncoated and coated droplet generator. The images show the bottom of the channel near the outlet. The scale bar represents 500 µm (top) and 50 µm (bottom).

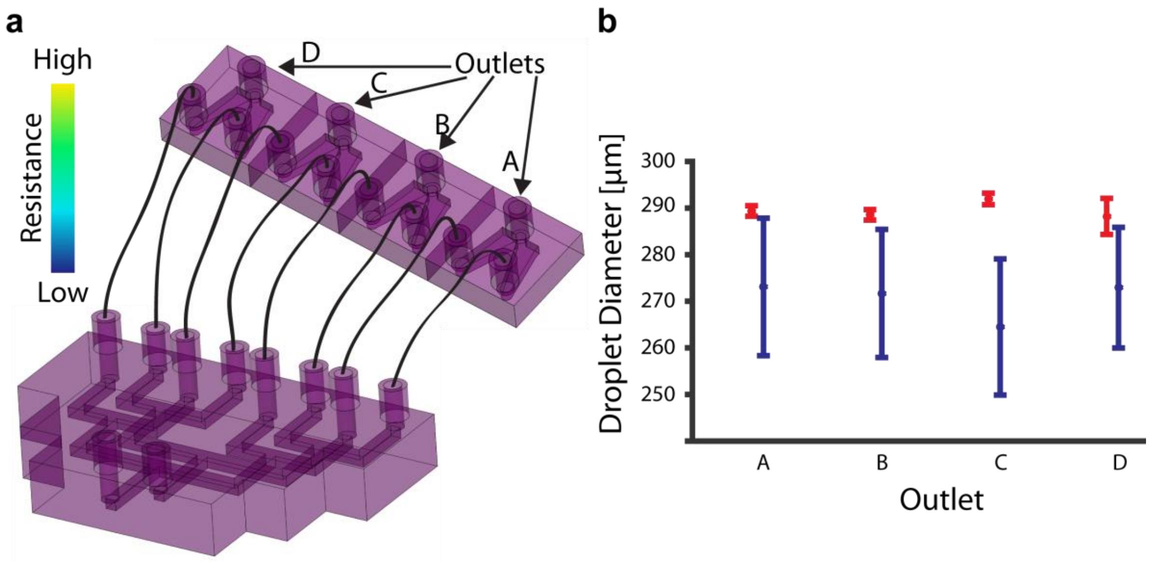

Supplementary Figure 7 Performance of balanced parallel network. The parallel network is set up to deliver equal flow rates to each of the four outlets. Both the flow invariant regime (red) and flow dependent regime (blue) produce the same size droplets across the four outlets when the pressure drop across the four droplet generators is balanced. The dispersed phase flow rate:continuous phase flow rate is 20:70 mL h^{-1} and 60:210 mL h^{-1} for the (red) and (blue) points, respectively. Error bars represent the s.d.

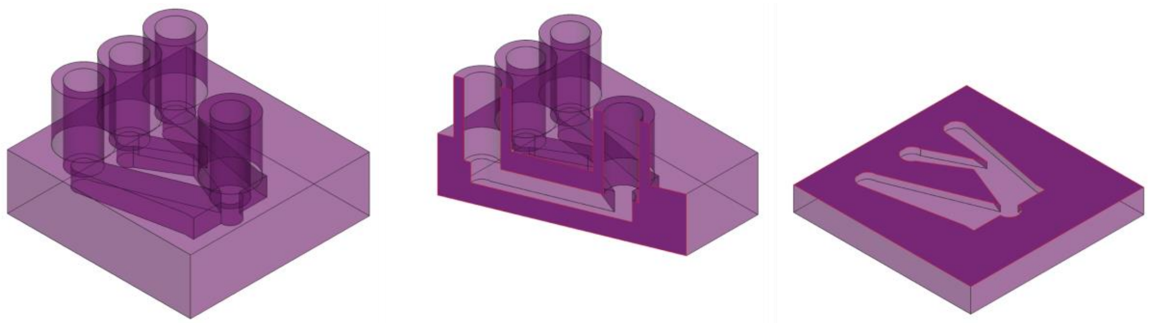

Supplementary Figure 8 Device configuration for PtNP synthesis. Rendering of the device geometry used for the synthesis of PtNPs. It contains one inlet for the continuous and two inlets for the dispersed phases and one outlet. Each port accepts OD = 1/16 inch tubing.

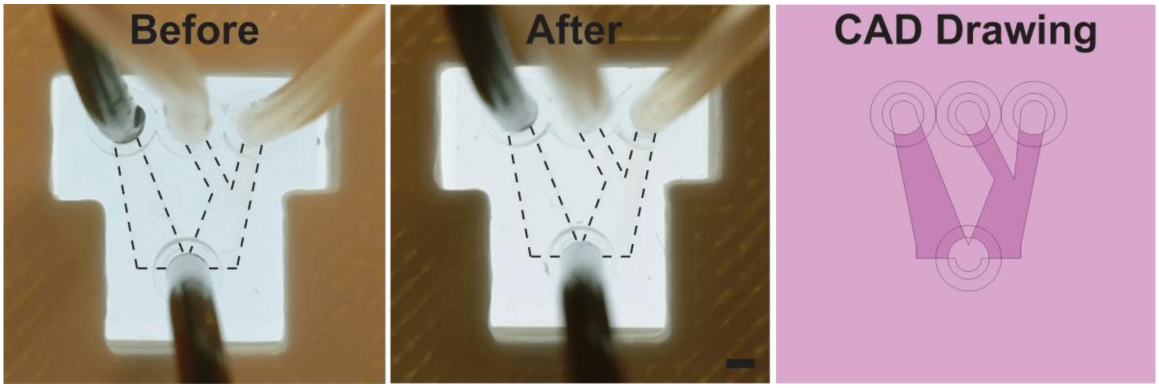

Supplementary Figure 9 Device resistance to fouling. Top down view of the droplet generating device from Supplementary Figure 8. It is held in place by a custom clamp. The droplet generating device remains visually unchanged after running the PtNP precursors. The CAD drawing shows the top-down view of the device. Scale bar is the same for all images and represents 1 mm.

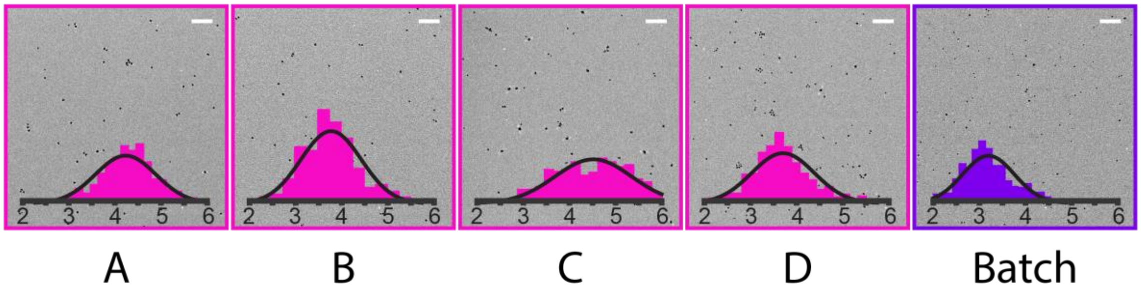

Supplementary Figure 10 Comparison of PtNPs. TEM micrographs of PtNPs produced in a parallel reaction from the four channels indicated in Supplementary Figure 7 and an analogous batch reaction. The histograms represent the population of 500 particles. The mean diameters for the parallel run are 4.23 ± 0.65 nm, 3.77 ± 0.66 nm, 4.52 ± 0.85 nm, and 3.71 ± 0.68 nm for outlets A through D, respectively. The Pt salt and IL streams are flown at 4x the single channel flow rates.

Supplementary Note

Supplementary Note 1 NMR Data.

<u>NMR of recycled BMIM-NTf$_2$</u>

Pure <u>BMIM-NTf$_2$</u>: ^{1}H NMR (600 MHz, $CDCl_3$) δ 0.94 (t, 3H, $NCH_2CH_2CH_2C\underline{H}_3$), 1.35 (m, 2H, $NCH_2CH_2C\underline{H}_2CH_3$), 1.83 (m, 2H, $NCH_2C\underline{H}_2CH_2CH_3$), 3.92 (s, 3H, $C\underline{H}_3N$), 4.15 (t, 2H, $NC\underline{H}_2CH_2CH_2CH_3$), 7.28 (d, 2H, $NC\underline{H}C\underline{H}N$) 8.74 (s, 1H, $NC\underline{H}N$)

<u>BMIM-NTf$_2$</u> recycled 1x: ^{1}H NMR (600 MHz, $CDCl_3$) δ 0.99 (t, 3H, $NCH_2CH_2CH_2C\underline{H}_3$), 1.40 (m, 2H, $NCH_2CH_2C\underline{H}_2CH_3$), 1.88 (m, 2H, $NCH_2C\underline{H}_2CH_2CH_3$), 3.98 (s, 3H, $C\underline{H}_3N$), 4.21 (t, 2H, $NC\underline{H}_2CH_2CH_2CH_3$), 7.27 (d, 2H, $NC\underline{H}C\underline{H}N$) 8.89 (s, 1H, $NC\underline{H}N$)

<u>BMIM-NTf$_2$</u> recycled 2x: ^{1}H NMR (600 ,MHz, $CDCl_3$) δ 0.99 (t, 3H, $NCH_2CH_2CH_2C\underline{H}_3$), 1.39 (m, 2H, $NCH_2CH_2C\underline{H}_2CH_3$), 1.88 (m, 2H, $NCH_2C\underline{H}_2CH_2CH_3$), 4.00 (s, 3H, $C\underline{H}_3N$), 4.22 (t, 2H, $NC\underline{H}_2CH_2CH_2CH_3$), 7.27 (d, 2H, $NC\underline{H}C\underline{H}N$) 8.90 (s, 1H, $NC\underline{H}N$)

^{1}H NMR was taken of the pure <u>BMIM-NTf$_2$</u> ionic liquid as well after recycling 1 and 2x. The resulting spectra matched literature assignments (Dunn, M.; Cole,M. L.; Harper, J. B. *RSC Adv.* **2012**, *2*, 10160-10162). Residual ethylene glycol was observed at δ 3.76 for the recycled ionic liquids.
